# Conversion of yellow wine lees into high-protein yeast culture by solid-state fermentation

**DOI:** 10.1080/13102818.2014.962407

**Published:** 2014-10-22

**Authors:** Yuanliang Hu, Lina Pan, Yaohao Dun, Nan Peng, Yunxiang Liang, Shumiao Zhao

**Affiliations:** ^a^ State Key Laboratory of Agricultural Microbiology, College of Life Science and Technology, Huazhong Agricultural University, Wuhan430070, P.R. China; ^b^ College of Life Sciences, Hubei Normal University, Huangshi, Hubei435002, P.R. China

**Keywords:** protein feed, *Saccharomyces cerevisiae*, solid-state fermentation (SSF), yeast culture, yellow wine lees

## Abstract

This study is focussed on the possibility of producing a yeast culture with yellow wine lees as a substrate by solid-state fermentation (SSF). Results showed that a yeast count of 1.58 × 10^9^ CFU/g was achieved by signal factor and orthogonal experiments. After fermentation, the starch content in the yeast culture reduced from 32.2% ± 0.5% to 7.5% ± 0.2%, and the contents of crude protein and peptide increased from 36.1% ± 0.8% to 48.0% ± 1.0% and 3.9% ± 0.2% to 7.2% ± 0.4%, respectively. Additionally, large amounts of short peptides and free amino acids were detected by fast protein liquid chromatography (FPLC). These results suggest that yellow wine lees are a suitable substrate for the production of yeast cultures. It can serve as a growth-promoting factor and help reduce the shortage of protein feed in the animal industry. This research provides a potential way for the utilization of agro-industrial residues.

## Introduction

Currently, a large amount of protein feed is needed to sustain the development of animal production. However, the high cost, and sometimes, the unavailability of commercial protein supplements, is one of the main limitations for animal production.[1] The shortage of protein feed is a worldwide problem. For example, China imported 58.38 million tons of soybeans and 1.3 million tons of fish meal in 2012 (the data are from the Ministry of Commerce of PRC). On the other hand, large quantities of low-value proteins, such as yellow wine lees, mezcal vinasses,[2] and brewer's spent grain [3] are produced worldwide in food production. They are similar in chemical composition and have not been fully exploited.[4] Yellow wine, one of the three most ancient wines in the world, is typically fermented from sticky rice.[5] In 2011, yellow wine production was 3.1 billion litres, and generated about 1 million tons of wine lees in China.[6] Usually, they are considered to be highly polluting because their discharge may have a negative impact on the ecosystems.

Yeasts such as *Saccharomyces cerevisiae* and *Candida utilis* have the ability to upgrade low-value protein by-products, such as cheese whey and molasses,[7] pineapple waste [8] and waste Chinese cabbage,[9] to high-protein materials – a supplement for animal feed. The bioconversion process to convert agro-industrial wastes into a valuable protein supplement for animal feed is generally thought to be an attractive way to both enhance waste treatment and increase resource utilization.[10] Yeast cultures that are produced through yeast fermentation contain fermentation by-products and are not dependent on live yeast for their physiological effects, or rather, the fermentation products contain compounds (mainly metabolites and polysaccharides), which could create suitable conditions for cellulolytic degradability and lactate usage by stimulating the development and activity of rumen bacteria, thus improving the growth performance and health of animals.[11,12] Currently, some yeast cultures are allowed to go on sale as feed additives, such as Diamond ‘XP’ (Diamond V Mills, Cedar Rapids, USA), which has been widely used in animal production, and achieved significant results.[13]

However, few reports are available about the conversion of agro-industrial residues into yeast culture. The objective of the current study was to investigate the possibility of using low-cost yellow wine lees as a substrate to produce a high-protein yeast culture, which could serve as functional feed additive and protein feed. It provided a potential way for the utilization of agro-industrial residues.

## Material and methods

### Strains

A commercial high-temperature resistant and highly active dry yeast was donated by Angel Yeast Company (Yichang, China), which contained *S. cerevisiae* 1 × 10^10^ CFU/g.

### Yellow wine lees and medium

Fresh yellow wine lees were donated by Chuyuanchun Winery Company (Yichang, China). On the same day, they were dried at 45 °C, sealed in plastic bags and then stored at 4 °C. YPD agar (%, w/v; glucose 2.0, peptone 2.0, yeast extract 1.0 and agar 1.8) was autoclaved at 115 °C for 20 min. Before use, the medium was supplemented with ampicillin and the final concentration was 100 μg/mL.

### Optimization of growth parameters

Initial fermentation conditions were as follows: the solid medium containing 30 g of dry yellow wine lees (dispensedin 360 mL jar) was inoculated with yeast 1 × 10^8^ CFU/g, with a water-to-material ratio of 1:1, and a natural pH value. All of the jars were incubated at 30 °C for 72 h. Then the samples were used for the assay of the yeast count. Single-factor experiments were carried out in terms of glucoamylase (Yuanye Biotechnology Company, Shanghai, China), the ratio of water to material, inoculum size, temperature, carbon sources and nitrogen sources. After that an orthogonal design in three levels of three factors (glucoamylase, soluble starch and (NH_4_)_2_SO_4_) was used for the experiments in order to obtain an optimum medium.

### Yeast count

After fermentation, the samples were removed from the jar, cut into similar size particles and dried at 45 °C. Then 10 g of the dry samples were dissolved in a 250 mL flask with 90 mL of sterile saline water (0.9%) and some sterile glass beads by shaking for 30 min (30 °C, 200 r/min). Finally, total yeast count was determined by a plate counting method (YPD agar; the gradient dilution from 10^−5^ to 10^−7^; 30 °C, 48 h).

### Yeast autolysis

In order to release the nutrients of the yeast cell after fermentation, the products in the jar (initial yellow wine lees 30 g) were supplemented with 45 mL of water, 180 mg of SUKAPro NE neutralprotease (50 U/mg; Sukahan, Weifang, China) and 12 mg of lyticase (400 U/mg; Pangbo Biotechnology Company, Nanning, China). Subsequently, they were held at pH 6.5 and 50 °C for 24 h before drying at 45 °C.

### Chemical composition analysis

Samples were analysed in crude protein, crude fibre, ether extract, reducing sugar and starch by following the procedures issued by AOAC INTERNATIONAL.[14] Briefly, the samples were ground into powder and passed through a 1-mm sieve before analysis. Then the crude protein content was determined by the micro-Kjedahl method. Crude fibre was measured by the intermediate filtration method and ether extract by extraction with petroleum ether (Soxhlet method). Reducing sugar amount was determined by the 3,5-dinitrosalicylic acid (DNS) method. For determining the starch content, the samples were degraded by amylase before the reducing sugar was measured. The peptide was assessed by the Biuret's method.[15]

### Molecular-weight determination by fast protein liquid chromatography (FPLC)

After drying, 1 g of yeast culture was dissolved in 10 mL of sodium acetate-acetic acid buffer (0.05 mol/L, pH 4.5), shaken well, precipitated for 10 min and centrifuged for 10 min (8000 g, 4 °C). The molecular-mass distribution of proteins in the supernatant was determined on a Superdex^TM^ 75 column (GE Amersham, Uppsala, Sweden) by size-exclusion chromatography using an AKTA FPLC system (GE Amersham).[16] The column was equilibrated, and eluted with 0.05 mol/L sodium acetate-acetic acid buffer (pH 4.5) in isocratic mode, at a flow rate of 0.4 mL/min. The molecular-weight distribution of yeast culture was determined after calibration of the column with standard proteins using Gel Filtration Standard, Catalogue No. 151-1901 (Bio-Rad, Hercules, USA).

### Statistical analysis

Each parameter was measured at least in triplicate. Conventional statistical methods were used to calculate means and standard deviations. Statistical significance was determined by analysis of variance and subsequent *F*-test (*P* < 0.05). The analysis was performed using SPSS (Version 19; Chicago, USA).

## Results and discussion

### Composition analysis of yellow wine lees

The crude protein in the yellow wine lees was the main component with a content of up to 36.1% ± 0.8%, followed by starch, with a content of 32.2% ± 0.5% (Table 1). The contents of other substances were ether extract 7.3% ± 0.3%, crude fibre 4.8% ± 0.2%, peptide 3.9% ± 0.2% and reducing sugar 3.0% ± 0.1%. The results indicated that the rich nutrition in yellow wine lees, and a substantial amount of the starch in rice, had not been used during the process of yellow wine fermentation. It is reported that waste from the food industry often contains a considerable amount of fermentable sugars that could be converted using microbes into high value-added products.[17]

**Table 1.** Chemical composition (%*) of yellow wine lees and yeast culture.

Item	Starch	Crude protein	Ether extract	Crude fibre	Peptide	Reducing sugar
Yellow wine lees	32.2 ± 0.5	36.1 ± 0.8	7.3 ± 0.3	4.8 ± 0.2	3.9 ± 0.2	3.0 ± 0.1
Yeast culture	7.5 ± 0.2	48.0 ± 1.0	9.8 ± 0.4	4.6 ± 0.3	7.2 ± 0.4	10.9 ± 0.3

*The average value of three samples with standard deviation.

### Single-factor experiments

**Figure 1.**
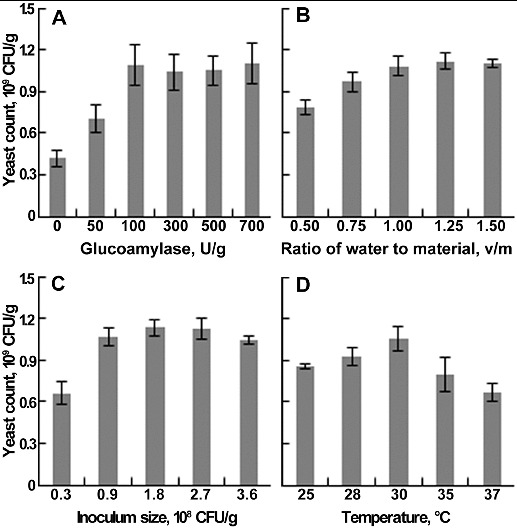
Effects of glucoamylase supplementation (A), ratio of water to material (B), inoculum size (C), and temperature (D) on yeast count. Each parameter was tested at least in triplicate. Error bars represent the standard deviation of the mean.

The yeast count was significantly increased by supplemented glucoamylase (Figure 1(A)). When the content of glucoamylase reached 100 U/g, the yeast count was 1.09 × 10^9^ CFU/g, 2.6-fold of the control. There was no obvious increase in yeast count with a glucoamylase content from 100 to 700 U/g. Therefore, 100 U/g was selected as the optimum amount of glucoamylase. Glucoamylase can hydrolyse starch almost completely into glucose, and its supplementation promoted the degradation of starch in wine lees, especially at the beginning of fermentation.[18] Moreover, yeast could produce large amounts of amylase to sustain the degradation and growth rate.

Moisture content is a crucial factor in solid-state fermentation (SSF), which affects the physical properties of the solid substrate, growth of microbes and the success of the bioprocess.[19] The effect of the water-to-material ratio (v/m) on yeast count (Figure 1(B)) showed that, at a ratio from 0.5 to 1, the yeast count increased linearly, and the maximal count was 1.08 × 10^9^ CFU/g. As the ratio reached 1.25, the yeast count increased slightly to 1.12 × 10^9^ CFU/g. As the ratio continued to rise, the yeast count decreased. The yeast count remained almost constant at a water-to-material ratio of 1 and 1.25; thus, 1 was determined as the optimum ratio. High moisture causes decreased porosity, lower oxygen transfer and alteration in substrate structure, while low moisture decreases the solubility of the solid substrate, lowers the degree of swelling and reduces solubility of the nutrients of the solid substrate.[19,20]

Inoculum size and temperature are the other two important factors influencing industrial fermentation, including lag phase duration, specific growth rate and biomass yield.[21,22] The maximal yeast count (1.14 × 10^9^ CFU/g) was obtained with an inoculum size of 1.8 × 10^8^ CFU/g, while at an inoculum size of 0.9 × 10^8^ CFU/g, the yeast count was 1.06 × 10^9^ CFU/g, showing that even with the inoculum size doubled, the yeast count was similar (Figure 1(C)). Considering the cost and benefit, 0.9 × 10^8^ CFU/g was selected as the optimum inoculum size. For temperature (Figure 1(D)), the results showed that the yeast count was the highest (1.05 × 10^9^ CFU/g) at 30 °C. As the temperature continued to rise, the yeast count decreased rapidly; hence, 30 °C was defined as the optimum temperature.

**Figure 2.**
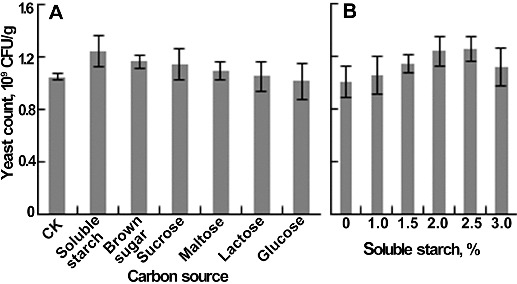
Effects of carbon source on yeast count. (A) Different carbon sources. (B) The content of soluble starch. Each parameter was tested at least in triplicate. Error bars represent the standard deviation of the mean

Several carbon and nitrogen sources were added into yellow wine lees to improve the yeast growth. In the experiment of carbon sources, the medium without carbon source supplementation served as control, and carbon sources (1%, w/w) were first mixed with water and then introduced into the lees. Among six kinds of carbon sources, soluble starch had the highest yeast count, followed by brown sugar (Figure 2(A)). Therefore, the soluble starch was selected as the supplemental carbon source. Further study showed that when the content of soluble starch was below 2%, the yeast count increased quickly, then slowly, and when the content reached 3%, the yeast count decreased (Figure 2(B)). The yeast count showed no significant difference at a soluble starch content of 2% and 2.5%, and thus 2% was determined as the optimum content.

**Figure 3.**
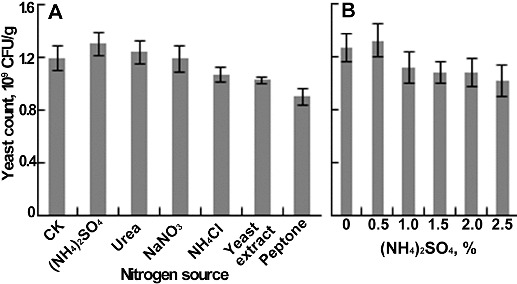
Effects of nitrogen source on yeast count. (A) Different nitrogen sources. (B) The content of (NH_4_)_2_SO_4_. Each parameter was tested at least in triplicate. Error bars represent the standard deviation of the mean.

In the nitrogen source experiment, the nitrogen (1%, w/w) was first mixed with water and then added into the lees. The result showed that the yeast count increased with the addition of (NH_4_)_2_SO_4_ or urea; however, a negative effect occurred with the addition of NaNO_3_, NH_4_Cl, yeast extract or peptone (Figure 3(A)). Thus, (NH_4_)_2_SO_4_ was selected to be studied further. As the content reached 1%, the number of yeast cells decreased rapidly, and 0.5% was found to be the optimum amount (Figure 3(B)).

In recent years, SSF has received more and more interest from researchers due to the advantages of high productivity, extended stability of products and low production costs.[23–25] SSF has been applied in various areas such as biotransformation of crops and crop residues for nutritional enrichment,[26] and the production of a range of high value-added products such as ethanol,[27] enzymes,[24] and single-cell protein.[7] As we know, one of the most important factors affecting the value of yeast products is biomass after fermentation, which can be measured by the yeast count. In single-factor experiments, the effect was found to be remarkable, especially for the optimization of glucoamylase. Furthermore, the soluble starch and (NH_4_)_2_SO_4_ were shown to be the best carbon source and nitrogen source, respectively, both of which are cheap and easy to obtain. Therefore, three factors (glucoamylase, soluble starch and (NH_4_)_2_SO_4_) were selected for further study.

### Orthogonal experiments

Table 2 shows that the theoretical optimum culture medium for yeast growth is 300 U/g of glucoamylase, 2% of soluble starch and 1% of (NH_4_)_2_SO_4._ The *R*-values indicated that the effects of the three factors on yeast count were ranked as glucoamylase > (NH_4_)_2_SO_4_ > soluble starch. However, variance analysis showed no significant difference among their effects on the yeast count (Table 3).

**Table 2.** Visual analysis of orthogonal experiment.

Runs	A	B	C	Yeast (× 10^9^ CFU/g *)
1	100	1%	1%	1.18 ± 0.08
2	100	2%	2%	1.22 ± 0.08
3	100	3%	3%	1.01 ± 0.07
4	200	1%	2%	1.21 ± 0.10
5	200	2%	3%	1.30 ± 0.07
6	200	3%	1%	1.47 ± 0.09
7	300	1%	3%	1.33 ± 0.09
8	300	2%	1%	1.58 ± 0.11
9	300	3%	2%	1.49 ± 0.10
*k*_1_	1.14	1.24	1.41	
*k*_2_	1.33	1.37	1.31	
*k*_3_	1.47	1.32	1.21	
*R*	0.33	0.13	0.20	

Note: The factor codes A (glucoamylase, U/g), B (soluble starch) and C [(NH_4_)_2_SO_4_]. *k* is the mean value of every factor and level. *R* is defined by *R* = *k*_max_ – *k*_min_.

*The average value of three samples with standard deviation.

**Table 3.** Variance analysis of orthogonal experiment.

Variation source	d.f.	Sum of squares	*F*-value	*F*_0.05_
Glucoamylase	2	0.165	2.568	4.460
Soluble starch	2	0.025	0.389	
(NH_4_)_2_SO_4_	2	0.058	0.903	
Error	8	0.260		

The yeast count reached the maximum value of 1.58 × 10^9^ CFU/g under the optimum conditions (Table 2). The results showed a relatively high level. It has been reported that the yeast count in potato processing waste fermented by yeast (containing 25% solids), and mixed agro-industrial wastes fermented by *S. cerevisiae* were 0.64 × 10^9^ CFU/mL [17] and 3.6 × 10^9^ CFU/mL,[28] respectively; and the yeast count in yeast culture was 1.3 × 10^9^ CFU/g.[29]

### Composition analysis of the yeast culture

As shown in Table 1, the content of crude protein was increased from 36.1% ± 0.8% to 48.0% ± 1.0% after fermentation, which reached the recommended level of 40%–52% for fodder yeast.[30] As we know, protein content is one of the most important criteria for evaluating the value of yeast products. Previous studies reported that the content of crude protein in dried yeast products grown in agro-industrial wastes reached 55.3% [8] and 43%.[9] Similarly, we transformed yellow wine lees with a relatively low-protein concentration into yeast culture with a high-protein concentration, which might be used as animal protein material to alleviate the shortage of protein feed.

**Figure 4.**
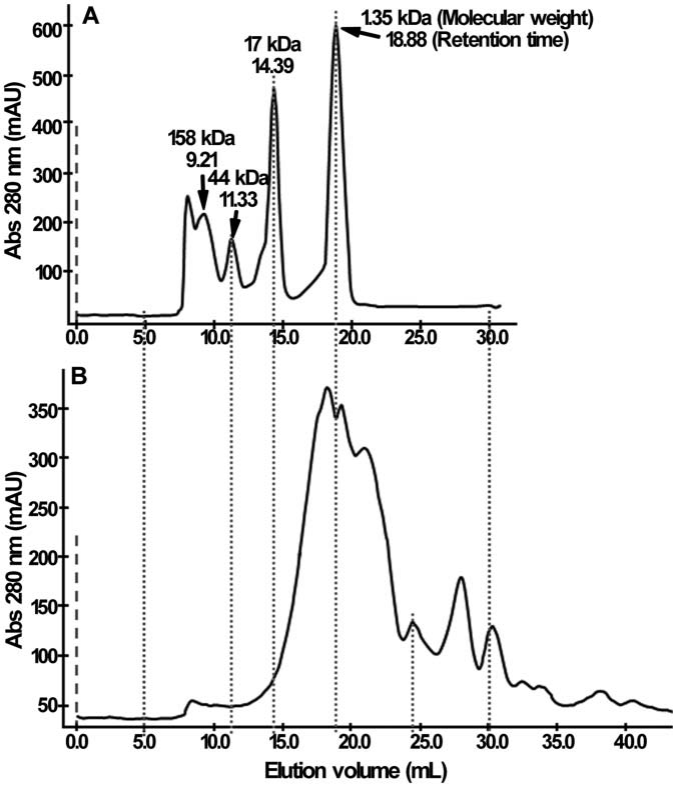
Molecular-weight distribution of acid soluble protein in yeast culture. (A) Standard protein. (B) Yeast culture. The size-exclusion chromatography was performed using a Superdex^TM^ 75 column (GE Amersham).

The content of peptides increased from 3.9% ± 0.2% to 7.2% ± 0.4% after fermentation (Table 1). In addition, for more than 50% of acid soluble proteins, the molecular weight was less than 1.35 kDa by FPLC (Figure 4), which suggested that the majority of soluble proteins in yeast cultures were short peptides and free amino acids. It has been reported that some peptides are highly desirable in nutrition and possess functional properties such as antibacterial, immunologic and antihypertensive effects, and could be used as functional compositions in food or in the feed formula.[31]

The crude starch content in the yeast culture reduced greatly, from 32.2% ± 0.5% to 7.5% ± 0.2% after fermentation (Table 1). A portion of the starch was hydrolysed to glucose during the fermentation process, and the reducing sugar increased from 3.0% ± 0.1% to 10.9% ± 0.3% (Table 1). Additionally, a large quantity of starch was eventually used by *S. cerevisiae*, and therefore, to some extent, the protein was concentrated and its content was increased. In fact, yeasts are frequently used for the production of microbial biomass, because of the ability to utilize a variety of carbon sources rapidly.[10]

## Conclusion

Generally, the yeast culture is produced by *S. cerevisiae* through the production processes of liquid-state fermentation (yeast proliferation), yeast autolysis (nutrients release), cereal grain adsorption and drying. The entire culture medium was retained in the product, without destroying the yeast factors, B-vitamins and other metabolites. In this study, SSF was used for the yeast proliferation, and multi-enzyme was used to promote the autolysis of yeasts and release their nutrients, and the subsequent drying was facilitated by the low-moisture content in the whole process. Therefore, we developed a potential method for producing yeast culture with yellow wine lees by SSF. After SSF and yeast autolysis, the content of crude protein and peptide increased, and large amounts of short peptides and free amino acids were detected by FPLC. These results suggest that yellow wine lees are a suitable substrate for the production of yeast culture, which might not only be used as protein feed, but also as a growth promoting factor in animal industry.
